# Co-infection of *Malassezia sympodialis* With Bacterial Pathobionts *Pseudomonas aeruginosa* or *Staphylococcus aureus* Leads to Distinct Sinonasal Inflammatory Responses in a Murine Acute Sinusitis Model

**DOI:** 10.3389/fcimb.2020.00472

**Published:** 2020-09-04

**Authors:** Keehoon Lee, Irene Zhang, Shari Kyman, Oliver Kask, Emily Kathryn Cope

**Affiliations:** Center for Applied Microbiome Sciences, The Pathogen and Microbiome Institute, Northern Arizona University, Flagstaff, AZ, United States

**Keywords:** malassezia, sinus microbiome, bacterial-fungal interactions, interkingdom interactions, *Staphylococcus aureus*, *Pseudomonas aeruginosa*

## Abstract

Host-associated bacteria and fungi, comprising the microbiota, are critical to host health. In the airways, the composition and diversity of the mucosal microbiota of patients are associated with airway health status. However, the relationship between airway microbiota and respiratory inflammation is not well-understood. Chronic rhinosinusitis (CRS) is a complex disease that affects up to 14% of the US population. Previous studies have shown decreased microbial diversity in CRS patients and enrichment of either *Staphylococcus aureus* or *Pseudomonas aeruginosa*. Although bacterial community composition is variable across CRS patients, *Malassezia* is a dominant fungal genus in the upper airways of the majority of healthy and CRS subjects. We hypothesize that distinct bacterial-fungal interactions differentially influence host mucosal immune response. Thus, we investigated *in vitro* and *in vivo* interactions between *Malassezia sympodialis, P. aeruginosa*, and *S. aureus*. The *in vitro* interactions were evaluated using the modified Kirby-Bauer Assay, Crystal Violet assay for biofilm, and FISH. A pilot murine model of acute sinusitis was used to investigate relationships with the host immune response. *S. aureus* and *P. aeruginosa* were intranasally instilled in the presence or absence of *M. sympodialis* (*n* = 66 total mice; 3–5/group). Changes in the microbiota were determined using 16S rRNA gene sequencing and host immune response was measured using quantitative real-time PCR (qRT-PCR). *In vitro*, only late stage planktonic *P. aeruginosa* and its biofilms inhibited *M. sympodialis*. Co-infection of mice with *M. sympodialis* and *P. aeruginosa* or *S. aureus* differently influenced the immune response. In co-infected mice, we demonstrate different expression of fungal sensing (Dectin-1), allergic responses (IL-5, and IL-13) and inflammation (IL-10, and IL-17) in murine sinus depending on the bacterial species that co-infected with *M. sympodialis* (*p* < 0.05). The pilot results suggest that species-specific interactions in airway-associated microbiota may be implicated driving immune responses. The understanding of the role of bacterial-fungal interactions in CRS will contribute to development of novel therapies toward manipulation of the airway microbiota.

## Introduction

Emerging studies of the microbiota, defined as bacteria, fungi, viruses, and archaea that inhabit a niche, have profoundly altered our understanding of the role of the human microbiota in health and disease. The range of effects that these complex microbial assemblages have on human physiology is unimaginably broad. For example, many studies suggest that gastrointestinal microbiota are related to not only intestinal diseases but also obesity (Moise, 2017), cancers (Gopalakrishnan et al., 2018), allergies (Fujimura et al., 2016), and neurological disorders (Elizabeth and Zabielski, 2013). In the gut, *Clostridium* produces the short-chain fatty acid (SCFA) butyrate, which contributes to the polarization of FOXP3+CD4+ regulatory T-cells. In addition to butyrate, gut microbes produce propionate and acetic acid, which can act as ligands for G protein-coupled receptors (GPCRs) and affect neutrophil chemotaxis, regulation of regulatory T cells (Treg), and dendritic cell (DC) maturation (Kau et al., 2011; Thaiss et al., 2016). However, our understanding of the respiratory tract microbiota remains nascent, even though it is one of the first body sites to make contact with microorganisms after birth and has a larger surface area than the skin or GI tract (Fröhlich et al., 2016). The development of culture-independent methods has shown that complex, site-specific microbial communities inhabit the upper and lower airways (Brill et al., 2016; Pletcher et al., 2018).

Chronic rhinosinusitis (CRS) is a common upper respiratory inflammatory disease affecting ~14% of the Western population and costing about $65 billion each year in disease management (Caulley et al., 2015). CRS is diagnosed through a combination of patient history, nasal endoscopy, and radiography and is characterized by symptoms lasting at least 12 consecutive weeks (Sedaghat, 2017). The primary goal of CRS treatments is to manage the symptoms and improve patients' quality of life. Medical management for CRS includes nasal saline irrigation (Gupta and Singh, 2010), topical intranasal and oral corticosteroids (Epperson et al., 2019), and antibiotics if a pathogen is cultured (Mattila, 2012). If medicinal management is unsuccessful, endoscopic sinus surgery is performed. Even though these treatment methods can improve symptoms, the recurrence rate of CRS is as high as 78.9% (Sedaghat, 2017; Calus et al., 2019).

The sinuses harbor a complex bacterial and fungal microbiome. A recent study from our group demonstrated that the sinus bacterial microbiota exists in four different compositional states each dominated by one of four different bacterial families and low-abundance co-colonizers. Each bacterial community state was associated with a distinct host inflammatory response in CRS patients (Cope et al., 2017). In another study using targeted qPCR, we demonstrated that *Malassezia* was the predominant fungal taxa across all patient subgroups (Gelber et al., 2016). Recent sequence-based studies have confirmed that *Malassezia* species, including *M. restricta* and *M. sympodialis*, are dominant core members of the sinus fungal microbiome (Carter and Amedee, 2014; Cleland et al., 2014; Hoggard et al., 2019; Wagner Mackenzie et al., 2019). *Malassezia* is a dimorphic and lipophilic fungus that is commonly found on the skin as a commensal organism. An opportunistic pathogen, *Malassezia* produces virulence factors such as lipases, phospholipases, and allergens that can damage epithelial integrity and trigger predominantly T_H_2 immune responses (Blanco and Garcia, 2008; Boekhout et al., 2010). In the GI tract, a study showed that *Malassezia* was associated with the colonic mucosa of Crohn's disease patients and exacerbated colitis in mouse models (Limon et al., 2019). However, the role of *Malassezia* in the respiratory tract has not been studied.

Interactions between bacteria and fungi are critical mediators of microbial community composition and function. Several studies investigating the interactions between bacteria and fungi have focused predominantly on another dimorphic yeast, *Candida albicans*, and bacterial co-colonizers. Distinct types of interactions not only affect the survival of the bacteria but also affect both fungal and bacterial virulence and host immune responses. For example, the interaction between *C. albicans* and *Staphylococcus aureus*, a bacterial pathogen, results in increased *C. albicans* hyphal formation and higher invasiveness of *S. aureus* into mucosal membranes by attaching to the hyphae of *C. albicans* (Schlecht et al., 2015). These studies of fungal and bacterial interactions suggest that we need to consider bacterial-fungal interactions to advance our understanding of chronic airway diseases such as cystic fibrosis (CF) and chronic rhinosinusitis (CRS) in the context of microbial interactions and relationships with the host immune response.

In this study, we hypothesized that bacterial co-colonizers, selected from our prior studies of the CRS bacterial microbiota, differentially interact with *Malassezia* and that this drives specific sinonasal immune responses dependent on innate immune sensing of fungi. Here, we investigated the *in vitro* and *in vivo* interactions between *Malassezia sympodialis* and bacterial pathogens, *Pseudomonas aeruginosa* and *Staphylococcus aureus*. *Malassezia* is the predominant fungal genus in the sinuses (Gelber et al., 2016; Hoggard et al., 2019), and *S. aureus* and *P. aeruginosa* are predominant members of two of the four described bacterial community states in CRS (Cope et al., 2017), and they are commonly isolated bacteria in CRS patients (Zhang et al., 2015). We evaluated *in vivo* influence of bacterial-*Malassezia* co-infection using a murine model of sinonasal disease (Abreu et al., 2012). These pilot studies suggest that *in vitro* interactions between *S. aureus* or *P. aeruginosa* with *M. sympodialis* are highly dependent on the species of bacteria and co-infection of *M. sympodialis* with either *S. aureus* or *P. aeruginosa* yields distinct sinonasal immune responses.

## Materials and Methods

### Bacterial and Fungal Species

We used two bacterial species, which were previously demonstrated to be related to CRS endotypes, and one fungal genus that belongs to the dominant fungal genus in the nasal passage, *Malassezia*. The bacterial species used were isolated from CRS patients: *Pseudomonas aeruginosa* EC1 (Cope et al., 2016) and *Staphylococcus aureus*. Species IDs were confirmed by sequencing the full length 16S rRNA gene. The fungal species, *Malassezia sympodialis*, was gifted by Paal Anderson (University of Copenhagen).

### Bacterial and Fungal Growth Media

*P. aeruginosa* and *S. aureus* were incubated in Sabouraud Dextrose Agar (SDA, HIMEDIA®) and SD broth. The lawn of *M. sympodialis* was grown on an SDA plate overlaid with ~300 μl sterile olive oil. Olive oil was not used in broth media for any organism as it inhibited some aspects of microbial growth. SDA with olive oil was not an optimal media because it inhibited aspects of microbial growth. The co-cultured biofilm experiment, the preparation for fluorescence *in-situ* hybridization (FISH) staining, and the *in vivo* mouse experiment were prepared using different media. The *P. aeruginosa* and *S. aureus* cultures were prepared in brain heart infusion broth (BHIB, BD Difco^TM^). *M. sympodialis* were prepared in modified Dixon (mDixon) media. Briefly, the contents of the modified Dixon media are 36 g malt extract (MP Biomedicals), 10 g Bacto^TM^ peptone (Life Technologies), 10 ml Tween 40 (Sigma-Aldrich), and 2 ml glycerol (Sigma-Aldrich) added to 1 L dH_2_O before pH was adjusted to 6.0 (Guillot et al., 1998). The desiccated ox bile and antibiotics were omitted from the original Dixon media in order to facilitate use in co-culture experiments with other bacterial species.

### *In vitro* Bacterial-Fungal Inhibitory Relationship Assay

We performed a modified Kirby-Bauer test to evaluate antagonistic relationships between *M. sympodialis* and *S. aureus* or *P. aeruginosa*. Each filter paper disc contained four different growth modes of *P. aeruginosa* or *S. aureus* culture (early phase planktonic: incubate for 3 h at 37°C with shaking; late phase planktonic: incubate for 24 h at 37°C with shaking). Growth discs containing biofilms were incubated for 24 h at 37°C in a 24-well plate. Biofilm discs were removed from the 24-well plate and immediately used in this assay. Finally, discs were inoculated with the cell-free supernatant fluid from late-phase planktonic bacterial growth media. The cell-free supernatant was prepared by filter-sterilizing the culture-supernatant after centrifugation at 8,000 × g for 1 min using a 0.22 μm syringe filter (MilliporeSigma^TM^ Millex^TM^). Discs were placed on the *M. sympodialis* lawn on Sabouraud Dextrose Agar (SDA) plates. The sterile Sabouraud Dextrose Broth (SDB) and 10% bleach solution on the discs were used for negative and positive growth inhibition controls, respectively. The plates were incubated at 30°C for 72 h, and the diameter of the inhibition zone around the discs was measured. This experiment was performed in triplicate.

### Crystal Violet Biofilm Assay

Primary broth cultures of *P. aeruginosa* or *S. aureus* were grown in brain heart infusion broth (BHIB, BD Difco^TM^) for 24 h at 37°C with shaking. 100 μl of the primary cultures were transferred into fresh 10 ml of modified Dixon (mDixon) and incubated for 4 h at 37°C with shaking to reach the exponential growth phase. The exponential growth phase cultures were diluted 1:100 in 150 μl of mDixon broth in a sterile 96-well plate (Cafarchia et al., 2012). To grow the *M. sympodialis* biofilm, the primary culture was grown in mDixon for 48 h at 30°C with shaking. 100 μl of the primary cultures were transferred into fresh 10 ml of mDixon and incubated for 6 h at 30°C with shaking to reach the exponential growth phase. The exponential growth phase cultures diluted 1:50 in 150 μl of mDixon in a sterile 96 well plates. For the polymicrobial biofilms, 1.5 ml of diluted the exponential growth phase culture of *M. sympodialis* and *P. aeruginosa* or *S. aureus* in mDixon were combined in a sterile 96 well plates. All the biofilm plates were grown at 30°C for 48 hrs without agitation. Biofilms were quantified using the crystal violet biofilm assay, as previously described (O'Toole, 2011). Briefly, the media from the 96 wells were discarded, and the wells were rinsed with distilled water and dried. 200 μl of 1% crystal violet staining solution was added to the wells to stain the biofilms for 10 min. The crystal violet staining solution was discarded and then the wells were rinsed with distilled water 3 times. The crystal violet stained biofilms were dried in the air for 3 h and 200 μl of 70% ethanol was added to extract the crystal violet stain from the biofilms. The amount of crystal violet stain in the 70% ethanol was measured by observing the optical density at 600 nm wavelength with (BioTek Synergy^TM^ HT). Uninoculated wells were used as negative controls. This experiment was repeated in quadruplicate.

### *In vivo* Murine Challenge Experiment

The *in vivo* model was modified from a previous study by Abreu et al. (2012). This experiment was performed once, and we used the same number of animals in this study per group (*n* = 3 mice/group for molecular analysis, *n* = 2 mice/group for Fluorescent *in situ* Hybridization as applicable) as previously published (Abreu et al., 2012). These studies were approved under NAU IACUC approval number 16-008. Mice were acclimatized for 5 days prior to the experiments. Two groups of balb/c mice (*n* = 66 total) were used: an antimicrobial-naive group (*n* = 3 mice/group for molecular analysis and *n* = 2 mice for fluorescent *in-situ* hybridization), and an antimicrobial-treated group (*n* = 3 mice/group for molecular analysis). The antimicrobial-treated mice were intranasally administered with augmentin (15 mg/kg) and fluconazole (100 mg/kg) for 5 days after a 5 day acclimatization period. Each group was intranasally infected with one either (1) saline (control), (2–4) single species microorganisms (1.0 × 10^8^ cells of *P. aeruginosa, S. aureus*, or *M. sympodialis*), or (5–6) dual-species microorganisms (1.0 × 10^8^ cells of *P. aeruginosa* and *M. sympodialis*, or *S. aureus* and *M. sympodialis*). The antimicrobial-naive and -treated mice were infected with the six infection groups mentioned above for 3 days. One day after the third infection, the mice were euthanized using CO_2_, and the sinonasal tissue was harvested and stored in 700 μl of RNAlater for microbiome and immunological analysis (Figure S1).

### Sinonasal Microbiome Analysis

The harvested sinonasal tissue samples in RNA*later*® (Sigma-Aldrich) were stored at 4°C for 24 h before processing the samples. After 24 h in RNA*later*®, DNA and RNA were extracted from the sinonasal tissue samples using the Allprep DNA/RNA mini kit (Qiagen). The V4 region of the 16S rRNA gene was amplified from DNA by polymerase chain reaction (PCR) using a 515f forward primer with an adapter sequence, AATGATACGGCGACCACCGAGATCTACACTATGGTAATTGTGTGCCAGCMGCCGCGGTAA, and the 806r indexed primers for reverse primers (Integrated DNA Technologies). The PCR reactions were carried out in 25 μL containing 1 μL of DNA template, 1x TaKaRa® Ex Taq PCR buffer (Mg2+ plus), 0.2 mM TaKaRa® dNTP mixture, 0.625 U TaKaRa® Ex Taq HS polymerase, 0.56 mg/μL BSA, 0.4 μM of each primer, and 16.375 μL of water. The PCRs were performed using a SimpliAmp® thermocycler (Applied Biosystems) under the following conditions: 98 °C for 2 min to release the polymerase antibody, followed by 30 cycles of 98°C for 20 s, 50°C for 30 s, and 72°C for 45 s. A final extension step of 72°C for 10 min was then conducted to ensure the completion of all fragments. All samples were triplicated and pooled in order to minimize the random amplification bias. The PCR amplicons were confirmed by agarose gel electrophoresis and quantified using Qubit 4 fluorometer (ThermoFisher Scientific). Amplicons were pooled at 50 ng/sample. The pooled amplicons were sequenced using Illumina MiSeq v3 (Illumina, inc.) at Translational Genomics North (TGen-North). The sequence data were analyzed using a bioinformatics platform, QIIME 2 (Bolyen et al., 2019).

### Murine Immune Gene Expression Analysis

The immunological responses were analyzed by measuring gene expression of the key immune responses in CRS (Baba et al., 2015; Hamilos, 2015; Hulse, 2016), such as genes for *interleukin (IL)-2, IL-5, IL-10, IL-13, IL-17, MUC5AC, Dectin-1*, and *Tumor Necrosis Factor (TNF)-*α, by quantitative reverse transcription polymerase chain reaction (qRT-PCR) using the 1 ng/μl of cDNA, which was synthesized from the extracted mRNA. Gene expression was normalized to glyceraldehyde-3-phosphate dehydrogenase (GAPDH) gene (Table 1). qRT-PCR was done using QuantStudio^TM^ 12k Flex Real-time PCR System (Applied Biosystems^TM^) with *Power* SYBR^TM^ Green PCR MasterMix (Applied Biosystems^TM^). The qRT-PCR condition consists of hold (50°C for 2 min and 95°C for 10 min.), PCR (40 cycles of 95°C for 15 sec. and 60°C for 1 min.), and melt curve cycle (95°C for 15 sec. and decrease to 60°C by 1.6°C /s).

**Table 1:** Primers used in this study.

**Primer**	**Sequence**	**Reference**
IL-2F	GTCACATTGACACTTGTGCTCC	Telander et al., 1999
IL-2R	AGTCAAATCCAGAACATGCCG	
IL-4F	TCGGCATTTTGAACGAGGTC	Kim et al., 2011
IL-4R	GAAAAGCCCGAAAGAGTCTC	
IL-5F	ATGGAGATTCCCATGAGCAC	
IL-5R	GTCTCTCCTCGCCACACTTC	
IL-10F	GCGTCGTGATTAGCGATGATG	Trandem et al., 2011
IL-10R	CTCGAGCAAGTCTTTCAGTCC	
IL-17F	GGACTCTCCACCGCAATGA	Atarashi et al., 2008
IL-17R	GGCACTGAGCTTCCCAGATC	
IFNg-F	CTACCTTCTTCAGCAACAGC	Oestreich et al., 2012
IFNg-R	GCTCATTGAATGCTTGGCGC	
GADPH-F	CCTCGTCCCGTAGACAAAATG	Ueda et al., 2010
GADPH-R	TCTCCACTTTGCCACTGCAA	
MUC5AC-F	AGCTACAGTGCAACTGGACC	Lin et al., 2014
MUC5AC-R	GGACACAGATGATGGTGACA	
IL13-F	AGGAGCTGAGCAACATCACAC	Kimura et al., 2014
IL13R	CCATAGCGGAAAAGTTGCTT	
TNF-α-F	GTAGCCCACGTCGTAGCAA	
TNF-α-R	AAATGGCAAATCGGCTGAC	
Dectin-1_F	ATCAGCATTCTTCCCCAACTCG	
Dectin-1_R	CAGTTCCTTCTCACAGATACTGTATGA	

### Fluorescence *in situ* Hybridization (FISH) of Polymicrobial Biofilms and Mouse Sinonasal Specimens

The bacterial pathogens, *P. aeruginosa* or *S. aureus*, and *M. sympodialis* co-developed biofilms were observed under the confocal laser scanning microscope (CLSM) using FISH. The biofilms were developed according to the protocol above. The biofilms were hybridized using the universal eubacterial probe, EUB 388, and *Malassezia* genus specific probe (Table 2). The biofilms were carefully transferred to glass slides and air-dried. The air-dried biofilms were fixed by dipping the slides in 50, 70, and 95% ETOH for 3 min each, and washed with DI water. The fixed biofilms were hybridized with 1 μl of the EUB 388 probe (10 nM) and 1 μl of the *Malassezia*_genus probe (10 nM) in 18 μl of the hybridization buffer for 90 min at 46°C in a hybridization chamber with damped paper with 5 ml of hybridization buffer for humidity. The hybridized biofilms were washed in a 48°C washing buffer for 30 min. The washed biofilms were rinsed briefly with DI water and mounted using VECTASHIELD® (VECTOR Laboratories). The hybridization buffer contained 0.9M NaCl, 20 mM Tris/HCl (pH 8.0), 0.01% SDS, and 35% formamide. The washing buffer contained 0.08M NaCl, 20 mM Tris/HCl (pH 8.0), 5 mM EDTA, and 0.01% SDS. Sinonasal specimens were prepared by fixing the skinned head of mice in methanol-Carnoy's fixative (60% methanol, 30% chloroform, 10% glacial acetic acid) for 24 h. Heads were decalcified in EDTA for 10 days, then embedded in a paraffin block. The micro-sectioned sinonasal tissue slide was prepared using the paraffin-embedded samples. The sinonasal tissue slides were deparaffinized by incubating the slide in xylene twice for 5 min each and washing it with 100% ethanol twice for 5 min each. The deparaffinized slides were hybridized using the same protocol with different probes. The probes used in the mouse sinonasal specimen FISH were 1 μl of 3 μM DAPI (4′,6-Diamidino-2-Phenylindole, Dihydrochloride), 1 μl of 10 nM *P. aeruginosa* specific probe (Paeru), 1 μl of 10 nM *S. aureus* specific probe (Saure), and 2 μl of 10 nM *Malassezia*_genus probe in 16 ul of the hybridization buffer (Table 2).

**Table 2:** FISH probes used in this study.

**Probe**	**Target**	**Fluor**	**Sequence 5^**′**^-3^**′**^**	**Reference**
EUB 388	Bacteria	Alexa 594	GCTGCCTCCCGTAGGAGT	Trebesius et al., 2000
Malassezia_genus	*Malassezia*	Alexa 532	CCGATATTTAGCTTTAGATGGAGTCTA	This study
Paeru	*P. aeruginosa*	Alexa 594	GGTAACCGTCCCCCTTGC	Hogardt et al., 2000
Saure	*S. aureus*	Alexa 660	GAAGCAAGCTTCTCGTCCG	Hogardt et al., 2000

### Bioinformatics and Statistical Analysis

For all comparisons, an α of 0.05 was considered significant. All *p*-values were corrected for false discovery using the Story method (Krzywinski and Altman, 2014). 16S rRNA gene sequences were analyzed using QIIME 2 version 2018.11 and 2019.1.0 (Bolyen et al., 2019); www.QIIME2.org). Sequences were demultiplexed and then truncated at 183 bases of the demultiplexed sequences to achieve a quality of >Q20 for all bases. Demultiplexed sequences were denoised and grouped into amplicon sequence variants (ASVs) using dada2 (Callahan et al., 2016). The feature table was rarified to a sampling depth of 1,134 sequences. A phylogenetic tree was built by aligning sequences with MAFFT and a phylogenetic tree was built using FastTree2 (Price et al., 2010; Katoh and Standley, 2013) which was subsequently rooted by midpoint rooting. Taxonomy was assigned to ASVs with a Naive Bayes classifier trained on the Greengenes 13_8 99% OTU database (DeSantis et al., 2006), using the q2-feature-classifier taxonomy classification plugin (Bokulich et al., 2018). Within-sample (α-diversity) was calculated using Pielou's Evenness index and a Kruskal Wallis test was used to compare across experimental groups. Between-sample (β-diversity) metrics were calculated using the UniFrac phylogenetic distance (Lozupone and Knight, 2005), Bray-Curtis, and Jaccard metrics (Goodrich et al., 2014). Permutational analysis of variance (PERMANOVA) between sinonasal communities of infection groups within antibiotic-treated and -naive groups was calculated on the Bray-Curtis dissimilarity matrix. The significance of the bacterial-fungal inhibitory relationship using the Kirby Bauer method was calculated using a paired *t*-test, and the Mann-Whitney *U*-test was used to compare the degree of biofilm development between the groups. For *in vivo* murine experiments, an unpaired *t*-test was used for comparisons of the murine immune gene expression.

## Results

### *In vitro* Bacterial and Fungal Interactions

**Figure 1:**
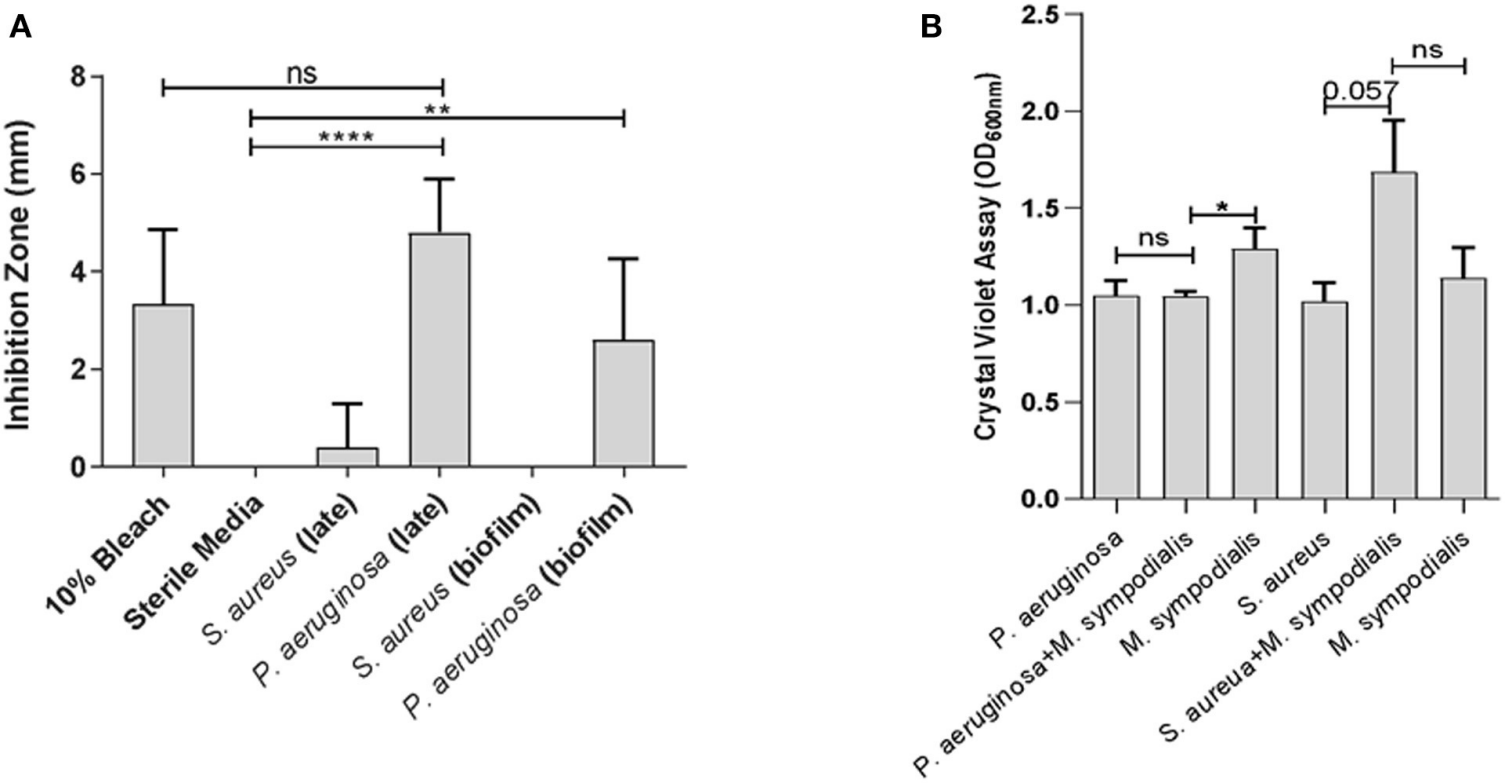
*In vitro* interactions between *Malassezia sympodialis* and different growth stages of *Pseudomonas aeruginosa* or *Staphylococcus aureus*. Zones of inhibition indicating antagonistic interactions in the modified Kirby-Bauer assay **(A)**, and quantification of biofilm development with *P. aeruginosa* and/or *M. sympodialis* or with *S. aureus* and/or *M. sympodialis* **(B)**. (ns, not statistically significant; *P ≤ 0.05, **P ≤ 0.005, ****P ≤ 0.00005).

To assess whether differential interactions between *M. sympodialis* and *S. aureus* or *P. aeruginosa* exist *in vitro*, we used a modified Kirby-Bauer inhibition assay and a crystal violet biofilm quantification assay. *Malassezia sympodialis* lawn was plated and paper discs inoculated with early phase planktonic, late phase planktonic, 24 h biofilm, and cell-free supernatant of the late phase planktonic culture were placed on the *M. sympodialis* lawn. We observed significantly larger inhibition zones when *P. aeruginosa* late phase planktonic and biofilm were applied to the discs compared to the control discs (paired *t*-test *p* = 0.0006 and 0.0255, respectively). The late phase planktonic *P. aeruginosa* and 10% bleach solution were not significantly different (Figure 1A, paired *t*-test, *p* = 0.4226). *S. aureus* did not inhibit *M. sympodialis* in any treatment group (Figure 1A, paired *t*-test *p* > 0.05). Biofilm formation was measured using the crystal violet assay for biofilm development. Dual-species biofilms of *P. aeruginosa* and *M. sympodialis* were significantly reduced compared to the *M. sympodialis* single-species biofilm (Figure 1B, Mann-Whitney *U*-test *p* = 0.0286). Dual-species *S. aureus* and *M. sympodialis* dual-species biofilms were slightly increased compared to either single-species biofilms but this did not reach statistical significance (Figure 1B, Mann-Whitney *U*-test *p* = 0.0571). In order to determine spatial organization of bacteria and *Malassezia in vitro*, dual-species biofilms were grown for 48 h and viewed using Fluorescent *in situ* Hybridization (FISH) and CLSM. The co-cultured biofilms of *M. sympodialis* with *S. aureus* or *P. aeruginosa* were stained using FISH probes EUB 388 and *Malassezia*_genus. *In vitro* FISH of the *M. sympodialis* with *P. aeruginosa* showed inhibition of *M. sympodialis* as we were only able to observe *P. aeruginosa* (Figure S2). However, there was clear colocalization of *S. aureus* and *M. sympodialis in vitro* (Figure S2). These results are the first to demonstrate species-specific interactions with a clinically relevant species of *Malassezia (M. sympodialis)* and two common airway pathobionts, *P. aeruginosa* and *S. aureus*.

### Sinonasal Microbiome Analysis

**Figure 2:**
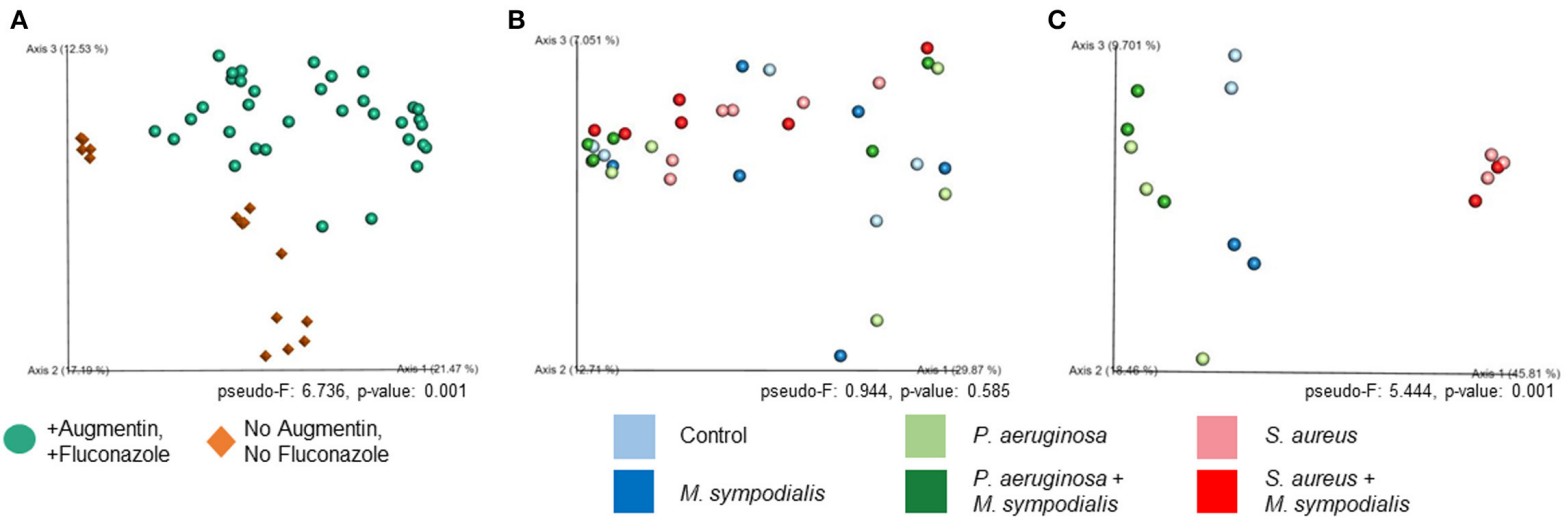
Principal Coordinates Analysis of Bray-Curtis Dissimilarity of the Murine Sinonasal Microbiome. Two groups of mice, antibiotic-treated or -naive, were intranasally infected with either one species of bacteria, *P. aeruginosa* (light green), *S. aureus* (light red), or fungi, *M. sympodialis* (dark blue), or two species, *M. sympodialis* with *P. aeruginosa* (dark green) or *S. aureus* (dark red). **(A)** represents beta-diversity of antibiotic-treated (solid sphere) and antibiotic-naïve (diamond) mice groups. **(B)** represents beta-diversity of the infection groups in the antibiotic-treated mice, and **(C)** represents beta-diversity of the infection groups in the antibiotic-naïve mice.

**Figure 3:**
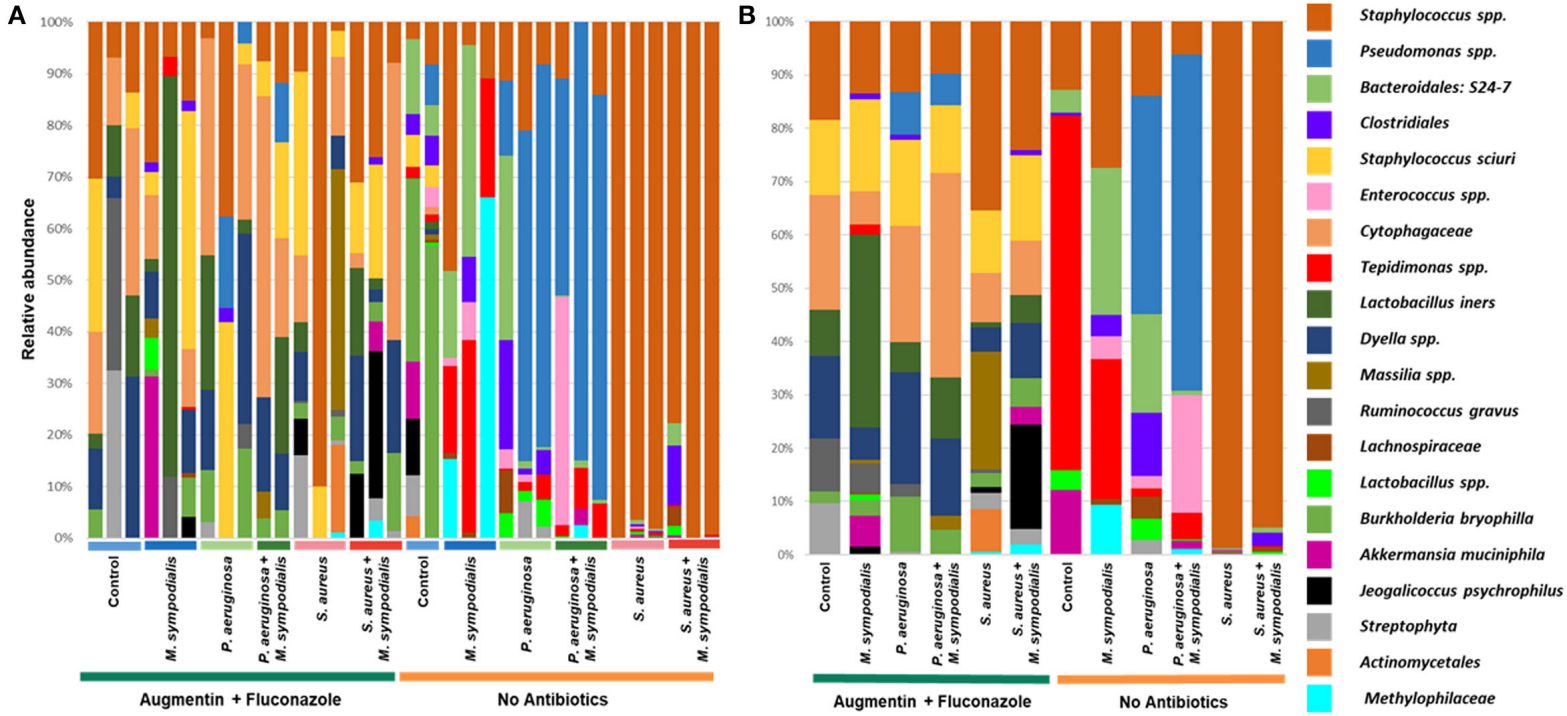
Relative bacterial abundances in sinonasal microbiota of mice. Two groups of mice, antibiotic-treated or -naive, were intranasally infected with either one species of bacteria, *P. aeruginosa* or *S. aureus*, or fungi, *M. sympodialis*, or two species, *M. sympodialis* with *P. aeruginosa* or *S. aureus*. The 20 most abundant taxa covers 90.494% of total sequence hits. **(A)** represents relative abundance of each sinonasal samples, and **(B)** represents the averaged relative abundances of each treatment group samples. The legend is an orderly representation of 20 most abundant bacterial taxa, top taxa being the most abundant.

We next sought to understand whether nasal instillation of *M. sympodialis* or bacterial pathobionts altered the bacterial sinonasal microbiome composition or diversity (*n* = 3 mice per group were used for microbiome analysis). We were interested in whether the introduction of a potential fungal pathobiont would significantly change the surrounding bacterial community members. We anticipated that nasal administration of *S. aureus* or *P. aeruginosa* would result in dominance of the respective taxa in both antibiotic-treated and antibiotic-naïve mice. Indeed, we found that introduction of bacteria, but not fungi, significantly altered the composition of the murine sinonasal bacterial microbiome. Between-sample diversity (beta-diversity) was significantly different between antibiotic-naïve and antibiotic-treated mice (Figure 2A, PERMANOVA, *p* = 0.001, Bray-Curtis Dissimilarity). There was no distinct separation between the infection groups of the antibiotic-treated samples (Figure 2B, PERMANOVA, *p* = 0.585), whereas the antibiotic-naïve mice samples clustered by the bacterial species inoculated (Figure 2C, PERMANOVA, *p* = 0.001). The *M. sympodialis* infected mice clustered with control mice and the co-infected mice clustered with the singly infected groups (Figure 2C), indicating that introduction of *M. sympodialis* does not significantly alter the murine sinonasal bacterial microbiome. We also classified taxonomy to examine relative abundances of taxa across groups. We have generated taxonomic barplots of relative abundance of 20 most abundant taxa overall which covers 90.494% of total taxa observed in our study (Figure 3). The taxonomic barpolts represent relative abundance of the top 20 taxa in sinonasal samples (Figure 3A) or the averaged relative abundances of each treatment group (Figure 3B). Taxonomic barplots show that the sinonasal microbiota was dominated by the bacterial species introduced intranasally only in the antibiotic-naïve mice (Figure 3 and Supplemental Information). Surprisingly, the composition of the sinonasal bacterial microbiota in the antibiotic-treated mice did not show obvious changes by the infected bacterial species as shown in the antibiotic-naïve groups (Figure 3, PERMANOVA, *p* = 0.001). The antibiotic-treated groups possess high portions of *Staphylococcus sciuri, Cytophagaceae, Lactobacillus iners*, and *Dyella* species, which were not detected in the antibiotic-naïve samples, and *Bacteriodalis*: S24-7 (*Muribaculaceae*) was detected only in the antibiotic-naïve group (Figure 3, interactive visualization in the supplemental information and on https://github.com/e-cope/malassezia-ms and can be viewed on view.qiime2.org). These results suggest that presence of *P. aeruginosa* and *S. aureus* on the sinonasal mucosa in antibiotic-naïve mice 1 day after intranasal instillation, but also show that *M. sympodialis* has a negligible effect on the sinonasal bacterial microbiota in this experiment.

### Analysis of the Sinonasal Immune Response

**Figure 4:**
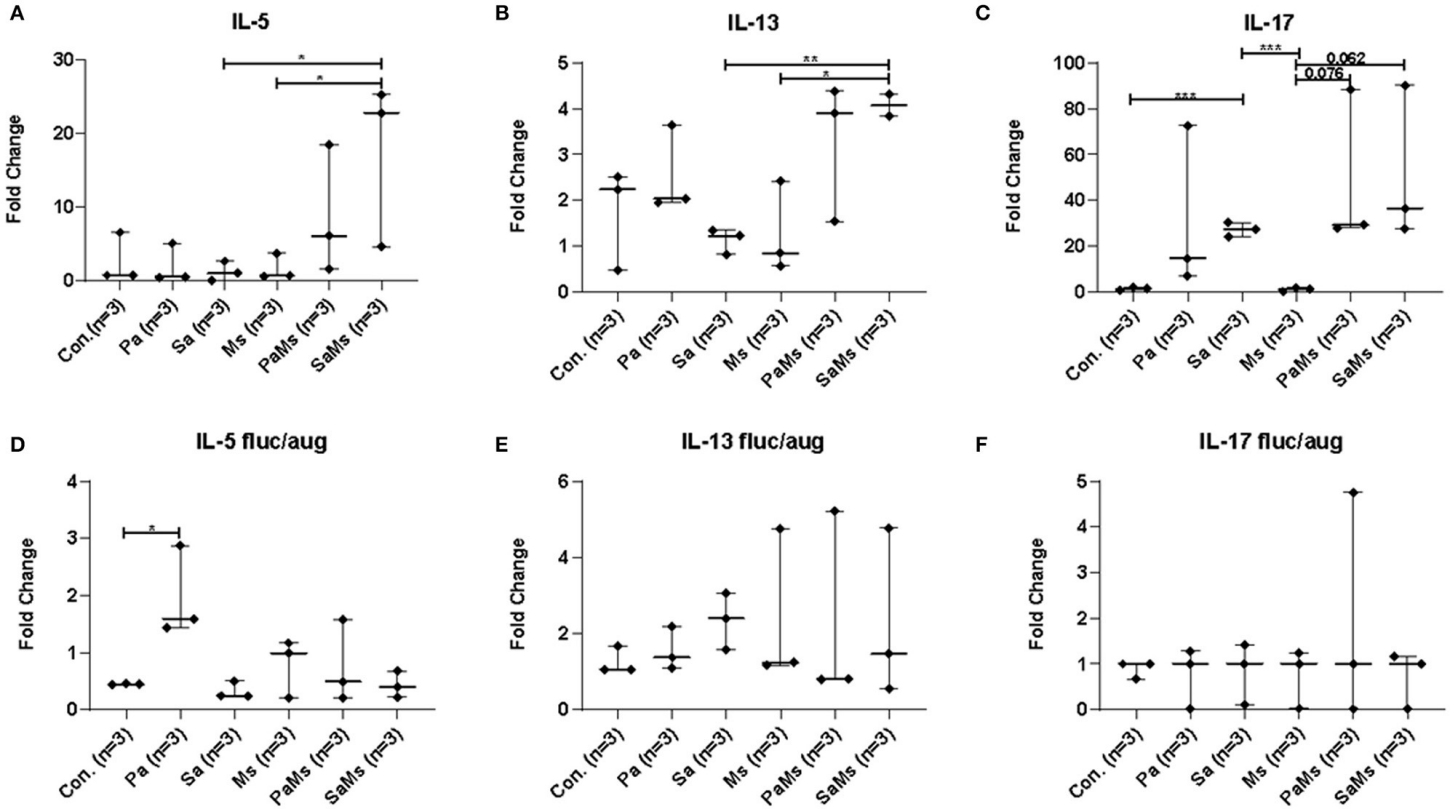
Relative gene expression of interleukin-5, interleukin-13, and interleukin-17 in the murine sinonasal cavity. The relative gene expression of IL-5 **(A,D)**, IL-13 **(B,E)** and IL-17 **(C,F)** in antibiotic-naïve **(A–C)** and antibiotic-treated **(D–F)** mice in different infection groups, saline treated group **(Con.)** *P. aeruginosa* infected group **(Pa)**, *S. aureus* infected group **(Sa)**, *M. sympodialis* infected group **(Ms)**, *P. aeruginosa* and *M. sympodialis* co-infected group **(PaMs)**, and *S. aureus* and *M. sympodialis* co-infected group **(SaMs)**, were measured by qRT-PCR. Gene expression was normalized with GAPDH, a housekeeping gene. Between-group comparisons without *p*-values were not statistically significant. (*P ≤ 0.05, **P ≤ 0.005, ***P ≤ 0.0005).

We next sought to determine whether co-infection with different combinations of *P. aeruginosa, S. aureus*, and *M. sympodialis* leads to distinct sinonasal immune responses. Gene expression of *IL-5, IL-13, IL-17, MUC5AC*, and *Dectin-1* were measured using reverse transcriptase quantitative PCR (*n* = 3 mice/group). *IL-5* gene expression significantly increased in *S. aureus* and *M. sympodialis* co-infected mice in antibiotic-naïve mice compared to control, *S. aureus* singly or *M. sympodialis* singly infected mice (Figure 4A, unpaired *t*-test, *p* = 0.0111, and *p* = 0.0188, respectively). However, *IL-5* gene expression was not elevated in antibiotic-treated mice co-infected with *S. aureus* and *M. sympodialis* (Figure 4D). *IL-5* expression significantly increased in the antibiotic-treated mice singly infected with *P. aeruginosa* (Figure 4D). *IL-13* gene expression had the same pattern as the *IL-5* gene expression. *IL-13* gene expression increased significantly in *S. aureus* and *M. sympodialis* co-infected, antibiotic-naïve mice compared to the *S. aureus* singly, or *M. sympodialis* singly infected mice (Figure 4B, unpaired *t*-test, *p* = 0.035, *p* = 0.0017, respectively). In antibiotic-treated mice, none of the infection groups had significant changes in *IL-13* gene expression (Figure 4E, unpaired *t*-test, *p* > 0.05).

*IL-17* gene expression significantly increased in *S. aureus* singly infected mice compared to controls (unpaired *t*-test, *p* = 0.0001) and *M. sympodialis* singly infected mice (unpaired *t*-test, *p* = 0.0001) in the antibiotic-naïve group. We observed a slight increase in increased in both co-infected mice, *P. aeruginosa* + *M. sympodialis* and *S. aureus* + *M. sympodialis* compared to the *M. sympodialis* singly infected mice in the antibiotic-naïve group but this was not statistically significant (Figure 4C, unpaired *t*-test, *p* = 0.061, *p* = 0.075). *IL-17* gene expression did not change in any of the infection groups when mice were treated with antibiotics prior to infection (Figure 4F, unpaired *t*-test, *p* > 0.05).

**Figure 5:**
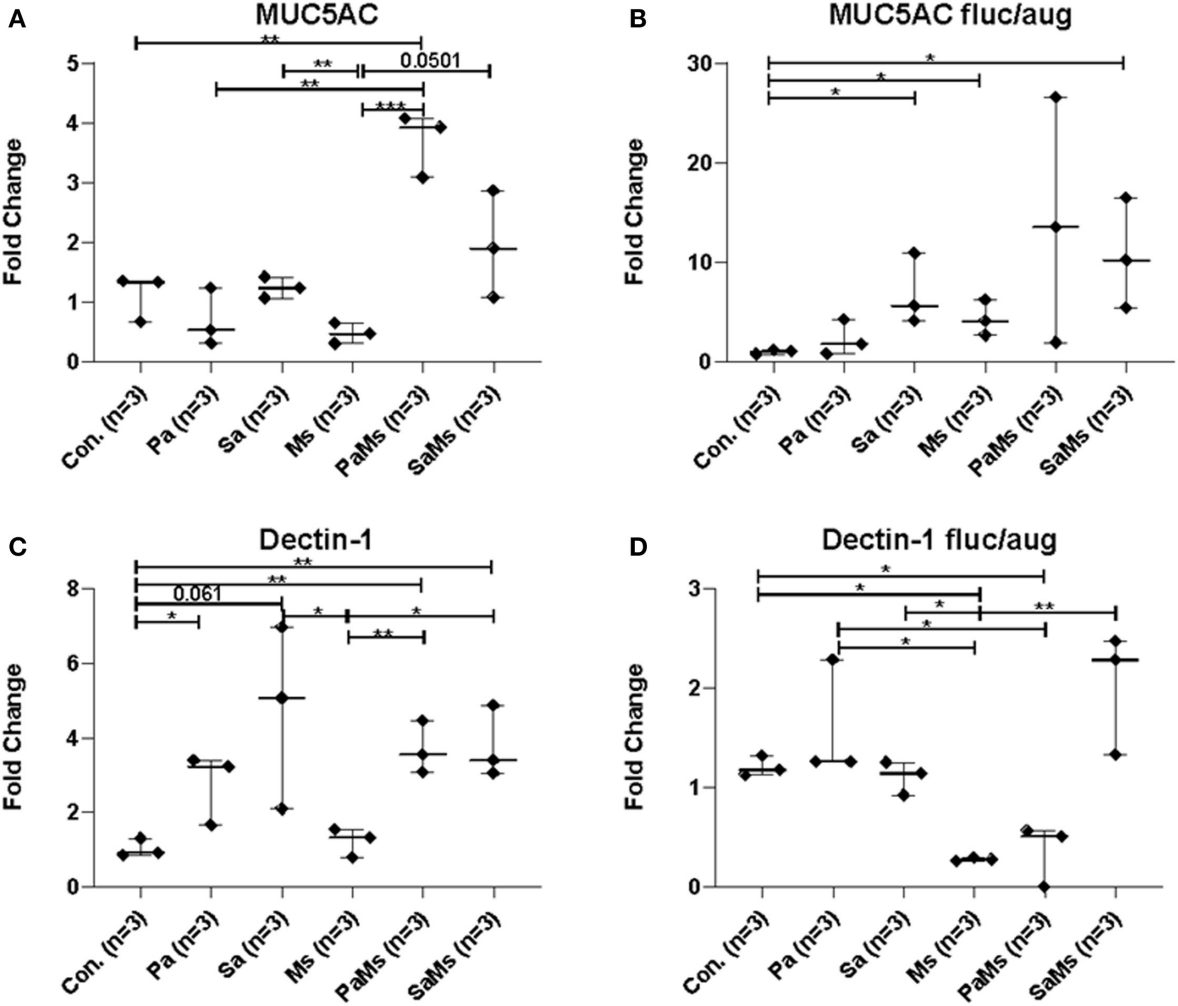
Relative gene expression of MUC5AC and Dectin-1 in the murine sinonasal cavity. Relative gene expressions of MUC5AC **(A,B)** and Dectin-1 **(C,D)** in antibiotic-naïve **(A,C)** and antibiotic-treated **(B,D)** mice on different infection groups, saline treated group **(Con.)** *P. aeruginosa* infected group **(Pa)**, *S. aureus* infected group **(Sa)**, *M. sympodialis* infected group **(Ms)**, *P. aeruginosa* and *M. sympodialis* co-infected group **(PaMs)**, and *S. aureus* and *M. sympodialis* co-infected group **(SaMs)**, were measured by qRT-PCR. The gene expression was normalized with GAPDH, a housekeeping gene. Between group comparisons without *p*-value were not statistically significant. (* *P* ≤ 0.05, ** *P* ≤ 0.005, *** *P* ≤ 0.0005).

*MUC5AC* gene expression significantly increased in antibiotic-naïve mice co-infected with *P. aeruginosa* and *M. sympodialis* when compared to control mice, *M. sympodialis* singly, and *P. aeruginosa* singly infected mice. We observed a slight but non-significant increase in *MUC5AC* gene expression in mice co-infected with *S. aureus* and *M. sympodialis* compared to mice singly infected with *S. aureus* or *M. sympodialis* (Figure 5A, unpaired *t*-test > 0.05). Of interest, *MUC5AC* gene expression was significantly decreased in *M. sympodialis* singly infected mice compared to *S. aureus* infected antibiotic-naïve mice (Figure 5A, unpaired *t*-test, *p* = 0.0059). In antibiotic-treated mice, *MUC5AC* was significantly increased in *S. aureus* singly, *M. sympodialis* singly infected mice, and *S. aureus* and *M. sympodialis* co-infected mice compared to control mice (Figure 5B, unpaired *t*-test, *p* < 0.05). We also evaluated the expression of *dectin-1*, a c-type lectin receptor involved in the recognition of *Malassezia* (Kistowska et al., 2014). *Dectin-1* expression significantly increased in *P. aeruginosa* singly infected, *P. aeruginosa* and *M. sympodialis* co-infected, and *S. aureus* and *M. sympodialis* co-infected mice compared to controls in the antibiotic-naïve group (Figure 5C, unpaired *t*-test, *p* < 0.05). *M. sympodialis* infection did not induce *dectin-1* gene expression, and *dectin-1* expression was not different between either co-infection group compared to the corresponding single bacterial infected mice (Figure 5C). In antibiotic-treated mice, *dectin-1* gene expression was not significantly different between control, *P. aeruginosa* singly infected, *S. aureus* singly infected, and *S. aureus* and *M. sympodialis* co-infected mice but it significantly decreased in *M. sympodialis* singly infected and *P. aeruginosa* and *M. sympodialis* co-infected mice compared to all other groups (Figure 5D, unpaired *t*-test, *p* < 0.05). In this pilot experiment, antibiotic-treated mice appeared to have a suppressive immune phenotype compared to antibiotic-naïve mice when infected with *P. aeruginosa, S. aureus*, or *M. sympodialis*. Future studies will confirm these findings in replicate experiments with additional mice.

### *Ex vivo* FISH on Murine Sinonasal Tissue

**Figure 6:**
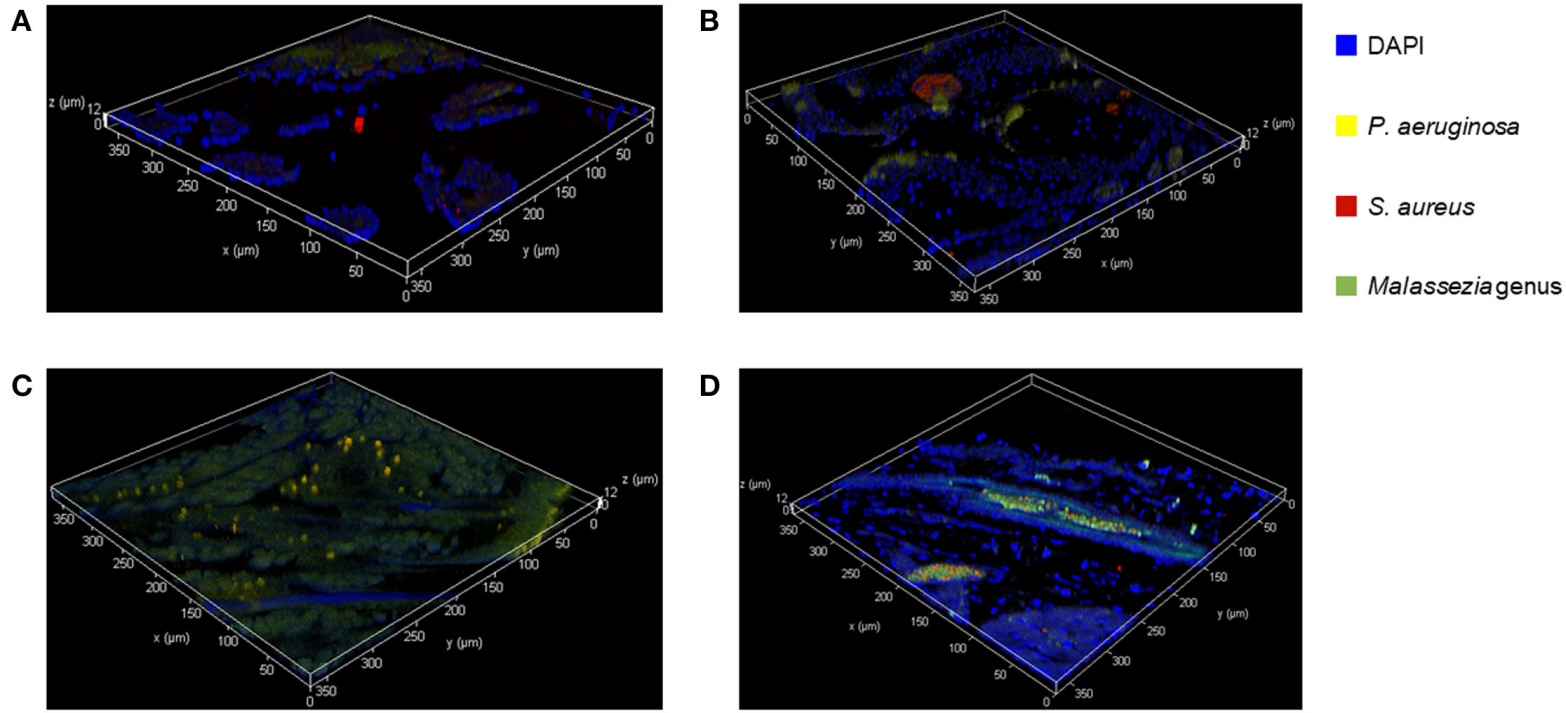
Fluorescent *in-situ* Hybridization (FISH) of the mice sinonasal tissues. Murine sinonasal tissues are hybridized with DAPI (blue) for tissue cells, and with FISH probes for *P. aeruginosa* (Alexa 594, pseudo-colored in yellow), *S. aureus* (Alexa 660, pseudo-colored in red) or genus *Malassezia* (Alexa 488, pseudo-colored in green). Hybridized specimens were observed using a confocal laser scanning microscope (CLSM) for 3 dimensional analysis. **(A)** Control, **(B)** *M. sympodialis* infected mouse, **(C)**, *M. sympodialis* and *P. aeruginosa* infected mouse, and **(D)** *M. sympodialis* and *S. aureus* infected mouse.

We used FISH to determine the spatial organization and confirm colonization of *M. sympodialis, P. aeruginosa*, and *S. aureus ex vivo* in the sinonasal cavity of mice that exhibited differential immune responses. *In vitro* FISH of the *M. sympodialis* with *P. aeruginosa* showed inhibition of *M. sympodialis* but there was clear colocalization of *S. aureus* and *M. sympodialis* (Figure S2). These results were confirmed in the sinonasal cavity of antibiotic-naïve mice after co-infection of each bacterial pathobiont with *M. sympodialis*. In control antibiotic-naïve mice, we observed *Malassezia* colonization and *S. aureus* biofilms, supporting our microbiome results (Figure 6A), and demonstrating the presence of *Malassezia* in the sinonasal microbiota. As expected, we observed increased *Malassezia* staining in mice singly infected with *M. sympodialis*. Endogenous *S. aureus* biofilms were also present, but we didn't observe strong colocalization of these two taxa in mice singly infected with *M. sympodialis* (Figure 6B). Notably, in mice co-instilled with *M. sympodialis* and *S. aureus* we demonstrate strong co-colonization of both microorganisms (Figure 6D). We also demonstrate potential intramucosal colonization of *M. sympodialis* and *S. aureus*, which may contribute to the differential immune response we observed in these mice. Intramucosal colonies of bacteria, most often *Staphylococcus* spp., have been observed in CRS patients, but the implications are unclear (Wood et al., 2012; Kim et al., 2013). Mice co-infected with *M. sympodialis* and *P. aeruginosa* had notably increased *P. aeruginosa* and less endogenous *S. aureus* colonies. We did not observe strong colocalization of *P. aeruginosa* with *Malassezia*, nor did we show a decrease in *Malassezia* genus (Figure 6C). These results show, for the first time, the spatial organization of *Malassezia* with bacterial pathobionts both *in vitro* and *ex vivo*.

## Discussion

Physical and chemical interactions between fungi and bacteria play important roles in various ecosystems, such as in biofilm infections (Schlecht et al., 2015), in the oral cavity (Janus et al., 2016), in soil (Warmink and van Elsas, 2009), or in food products (Kastman et al., 2016). These cross-kingdom interactions result in changes to microbial fitness and virulence levels (Shirtliff et al., 2009; Schlecht et al., 2015). In this study, we investigated the interaction between predominant sinonasal bacterial pathogens, *P. aeruginosa* and *S. aureus*, and a dominant fungus present in the upper respiratory tract, *M. sympodialis* (Findley et al., 2013; Cope et al., 2017). Our goal was to assess the influence of microbial interactions in the upper airway on bacterial microbiome composition and immune response.

Here, we present two main conclusions. First, we show that there are species-specific interactions *in vitro* between a predominant member of sinonasal mycobiota, *Malassezia*, and major pathobionts associated with CRS (*P. aeruginosa* or *S. aureus*), and that co-infections with *M. sympodialis* and *P. aeruginosa* or *S. aureus* leads to distinct immune responses. This conclusion is supported by the *in vitro* studies and the increased immune response in co-infected mice. The second conclusion is that an intact sinonasal microbiota may be required for inducing distinct immune responses mediated by bacterial-fungal interactions. This is also inferred from the immune response analyses which shows that mice with depleted sinonasal microbiota by antimicrobial treatment had a suppressed immune response to intranasal pathobiont challenge. These pilot results are limited by a small sample size in mice and represent a single experiment. Taken together, these results seem to indicate that resident microbiota, and not just the single bacterial pathobionts, may be critical in inducing an immune response. Future studies using a larger sample size, longitudinal, and multiple-'omics analyses are necessary to identify the directionality and mechanism driving interactions.

In order to understand the interactions between *S. aureus, P. aeruginosa*, and *M. sympodialis*, we performed a modified Kirby-Bauer inhibition assay, a crystal violet biofilm quantification assay, and *in vitro* Fluorescent *in situ* Hybridization (FISH). The results from the Kirby-Bauer inhibition assay indicated that the late growth phase and biofilm-mode of *P. aeruginosa* inhibit *M. sympodialis* growth. The early growth phase and the cell-free supernatant of late growth phase *P. aeruginosa* did not inhibit *M. sympodialis* (data not shown), suggesting that inhibition of *M. sympodialis* by *P. aeruginosa* may require direct cell-cell contact and the inhibition mechanism may be dependent on the density of *P. aeruginosa*. In addition, quorum sensing (QS) molecules, which can trigger changes in biofilm physiology and gene expression and require high microbial density, may influence the observed negative interactions between *P. aeruginosa* and *M. sympodialis*. One possible inhibitory mechanism between *P. aeruginosa* and *M. sympodialis* is the type VI secretion system (T6SS). T6SS is a QS-regulated nanomachinery system in bacterial membranes that allow the bacteria to inject toxins into other cell membranes or cytoplasm (Gallique et al., 2017). *P. aeruginosa* possesses 3 genes for T6SS, H1-, H2-, and H3-T6SS, and H2- and H3-T6SS act not only against prokaryotes but also against eukaryotes (Hood et al., 2010; Trunk et al., 2018). A recently published study showed that programmed inhibitor cells can target bacterial species in a complex microbial community using T6SS (Ting et al., 2020), indicating a potential role for T6SS in altering microbiota composition.

We demonstrated a commensal or synergistic interaction between *S. aureus* and *M. sympodialis*. We observed a small positive effect on biofilm development when *S. aureus* was co-cultured with *M. sympodialis* in contrast to decreased biofilm growth when *P. aeruginosa* and *M. sympodialis* were cultured together. The *in vitro* FISH images also demonstrated colocalization of *M. sympodialis* and *S. aureus* in a biofilm but not with *P. aeruginosa*. This indicates that there may be a positive interaction between *M. sympodialis* and *S. aureus*. Other studies have shown positive interactions between the dimorphic yeast, *Candida albicans*, and *S. aureus* (Shirtliff et al., 2009; Schlecht et al., 2015). In these studies, co-existence of *S. aureus* and *C. albicans* induced fungal hyphae formation, and *S. aureus* formed biofilm specifically on fungal hyphae. As a result of this interaction, the hyphal form of *C. albicans* penetrated into the host tissue to obtain nutrients, and *S. aureus* secured a niche to invade host tissue, which increased the virulence of both microbes (Schlecht et al., 2015). There also are studies that reported a high incidence of co-existence of *S. aureus* and *Malassezia* spp in dermatological diseases, such as seborrheic dermatitis, atopic dermatitis, and erythroceruminous otitis (Buda and Miedzobrodzki, 2016; Nuttall, 2016; Tamer et al., 2018). Thus, the interaction between *M. sympodialis* and *S. aureus* may also have an influence on the host immune response related to chronic rhinosinusitis and should be studied further. Furthermore, we performed FISH on sinonasal specimens in order to confirm how co-infection impacts the abundance and localization of *Malassezia* in mice. We demonstrated an increase in *Malassezia* genus abundance in sinonasal tissue of mice singly infected with *M. sympodialis*, but we did not observe colocalization of *Malassezia* with endogenous *Staphylococci*. Of interest, in mice co-infected with exogenous *Malassezia* and *S. aureus*, we observed colocalization of both taxa in the submucosa. Submucosal *S. aureus* has been observed in chronic rhinosinusitis, but the implications are unclear. In one study, epithelial invasion of *S. aureus* was associated with an elevated immune response (Sachse et al., 2010), and in other studies, submucosal *S. aureus* is associated with reduced eosinophilia (Wood et al., 2012). Overall, these pilot results support that the interactions we observed *in vitro* are maintained in an acute sinusitis model *in vivo*. Future studies will evaluate the spatial organization of the microbiota in murine sinus tissue using Combinatorial Labeling and Spectral (CLASI)-FISH in a larger sample size.

The sinonasal microbiome was altered by antibiotic use and infection. The bacterial composition of the antibiotic-naïve mice was driven by the infected bacterial species, though we did not observe the same effect in antibiotic-treated mice. For the microbiome analyses, we grouped sequences into Amplicon Sequence Variants (ASVs), or 100% OTUs using DADA2. This is a relatively new approach that allows higher resolution than clustering into 97% OTUs, as was the previous gold standard. This allows us to differentiate some native *Staphylococcus* taxa from those we instilled into the murine nasal cavity if they differ by a single nucleotide. However, There are still limitations to 16S rRNA amplicon sequencing, including an inability to distinguish between species (*Staphylococcus aureus* and *Staphylococcus epidermidis*), particularly using the V4 region of the 16S rRNA gene. We did not detect any endogenous *Pseudomonas* in any control sample, though other studies have detected *Pseudomonas* in the murine airways (Cope et al., 2016). This could be due to different strains and vivarium conditions across studies. Our microbiome results show that the sinonasal microbiota of mice infected with *P. aeruginosa* or *S. aureus* were dominated by the infected bacterial species regardless of the co-infection with *M. sympodialis*. Interestingly, *M. sympodialis* infection alone did not significantly alter the composition of the sinonasal bacterial communities. These results indicated that the murine sinonasal microbiota was altered by antibiotic treatments, and these changes impaired colonization by the newly infected bacteria. The pathobionts, *P. aeruginosa* and *S. aureus*, were introduced to the undisturbed sinonasal microbiota and successfully colonized, becoming the predominant bacterial genera present in the sinonasal microbiota. Future studies should include additional animals in replicate experiments to confirm these results.

Antibiotic treatment affected the sinonasal immune response after single- and co-infection. Augmentin and Fluconazole were used to examine the effect of single or co-infections without the bias of an intact sinonasal microbiota. In a prior study of acute sinusitis, Abreu et al. demonstrated that the depletion of native microbiota using Augmentin resulted in increased goblet cell hyperplasia after nasal instillation of the pathobiont *Corynebacterium tuberculostearicum* compared to antibiotic-naïve mice with an intact sinonasal microbiome (Abreu et al., 2012). For this reason, we decided to include an antibiotic-treated group and antibiotic-naïve group to compare immune responses when the sinonasal microbiota are intact or depleted. We observed no statistically significant changes between different infection groups in the antibiotic-treated mice with one exception of increased IL-5 gene expression in *P. aeruginosa*-infected mice. However, there were several statistically significant immune responses in the antibiotic-naïve mice, in which we also observed major changes to the sinonasal microbiome. The interaction between *S. aureus* and *M. sympodialis* may contribute to the induction of Th2 and Th17 immune responses. IL-5 and IL-13 were elevated in *S. aureus* and *M. sympodialis* co-infected mice. These cytokines are involved in Th2 immune response, leading to increased mucus production and allergic response (Pope et al., 2001). In CRS, IL-5 expression is a signature immune endotype, often associated with concurrent asthma or nasal polyps (Tomassen et al., 2016; Hoggard et al., 2018). MUC5AC expression was also elevated in co-infected mice, which is downstream of IL-13 gene expression. Interestingly, we observed the greatest increase in *M. sympodialis* and *P. aeruginosa-*infected mice in the presence of an intact sinonasal microbiome. IL-17 expression was elevated in *S. aureus* infected mice. Th17 is another signature of CRS inflammatory endotypes, and may contribute to polyp generation in human disease (Tomassen et al., 2016; Miljkovic et al., 2017). IL-17 is required for protection against *S. aureus* cutaneous infection, so the induction of IL-17 in the sinonasal cavity may be protective (Cho et al., 2010). Pulmonary infection with *S. aureus* also induces a prominent Th17 response in mice (Frank et al., 2012). Interestingly, IL-17 was repressed in antibiotic-treated mice, suggesting a role for the sinonasal microbiome in Th17 induction in the airways as has been demonstrated in the GI tract, dependent on colonization with Segmented Filamentous Bacteria (Ivanov et al., 2009). However, we did not observe a significant immune response in the antibiotic-naive mice singly infected with *M. sympodialis*, indicating that the endogenous *S. aureus* strain is either at a low abundance and thus insufficiently interacts with *M. sympodialis*, or that the endogenous *Staphylococci* have a different interaction with *Malassezia* compared to the pathobiont that we co-infected mice with. Importantly, we actually observed a suppressive effect when *M. sympodialis* was singly inoculated into the mice.

We were surprised that depletion of the sinonasal microbiota generally suppressed the immune response; this result was in contradiction to our hypothesis and to prior studies (Abreu et al., 2012; Cope et al., 2016). In the current study, we administered antibiotics intranasally, while prior studies have used oral antibiotics. Intranasal antibiotics may have had a more robust effect on the sinonasal mucosal microbiome. In addition, we depleted both the bacterial and fungal microbiota using augmentin and fluconazole, while other studies have only used antibiotics targeting bacteria. It's possible that depleting the sinonasal fungal microbiota along with the bacterial microbiota, we have inhibited the potential for colonization, or that the remaining microbiota (e.g., *Lactobacilli*) are inhibitory toward exogenous *M. sympodialis, S. aureus*, or *P. aeruginosa*. We don't think that there are residual antibiotics after 24 h, prior studies have shown that when Augmentin and Fluconazole are cleared from the system ~1 day after the oral or intravenous administration (Foulstone and Reading, 1982; Ripa et al., 1993). However, this is a possibility that we will address in future studies.

*Malassezia* has the ability to bind C-type lectin, a protein that recognizes fungal carbohydrates and can induce an immune response by binding to ligand (Graham and Brown, 2009). Studies of atopic eczema have shown that the cellular component of *Malassezia* affects the expression of a C-type lectin receptor gene, *dectin-1*, which results in aggravation of the disease, as measured by increased IL-6/IL-8 gene expression and β-hexosaminidase, histamine, and tryptase release (Ribbing et al., 2011). In both antibiotic-treated and antibiotic-naïve mice infected with *M. sympodialis* alone, we observed no increase in *dectin-1* gene expression. This was a surprising finding, but we also observed differential regulation of *dectin-1* in mice infected with *M. sympodialis* with bacterial co-colonizers. When mice were co-infected with *M. sympodialis* and *S. aureus, dectin-1* expression increased compared to *M. sympodialis* singly infected group, which may be due to increased *M. sympodialis* virulence mediated by the synergistic interaction with *S. aureus*. These results support our hypothesis that the interaction between *S. aureus* and *M. sympodialis* leads to a change in microbial community behavior, leading to increased fungal sensing. Future studies will seek to define the mechanisms underlying these findings. Dectin-1 activation can lead to increased Th2-type CD4+ T cell responses elicited by plasmacytoid dendritic cells, which could be one mechanism leading to elevated IL-5 and IL-13 gene expression in our study (Upchurch et al., 2016). Dectin-1 expression is also increased in CRS polyp tissue; future studies could evaluate whether this is correlated with co-colonization of *S. aureus* and *Malassezia* species (Gong et al., 2013). Interestingly, *dectin-1* expression also increased in antibiotic-naïve mice that were singly infected with bacteria (*S. aureus* or *P. aeruginosa*), but this was not observed in antibiotic-naïve mice singly infected with *M. sympodialis*. This could be due to bacterial interactions with endogenous *Malassezia* present in the murine sinonasal cavity or due to the sensing of bacterial ligands by Dectin-1, as has been demonstrated in the lungs with Dectin-1 sensing of non-typeable *Haemophilus influenzae* (Heyl et al., 2015).

This study has limitations. First, we are presenting findings based on a single pilot murine experiment of 3–5 animals per group. These numbers and replicates were based on published studies of acute sinusitis (Abreu et al., 2012; Cope et al., 2016) but we acknowledge that future studies should aim to replicate these findings in a larger sample size across different strains of mice. Thus, while exciting, these findings should be considered in the context of a small samples size in a single experiment. We plan to address these pitfalls in future studies that are based off these initial findings.

In conclusion, we demonstrate that interkingdom, species-specific interactions between a predominant member of sinonasal mycobiota, *Malassezia*, and major pathobionts associated with CRS (*P. aeruginosa* or *S. aureus*) leads to distinct immune responses at the sinonasal mucosa. *Malassezia* is an understudied fungal taxa with potential importance in a myriad of gut, skin, and respiratory disease (Hoggard et al., 2019; Limon et al., 2019; Ianiri et al., 2020). We also demonstrated that an endogenous sinonasal microbiota may be required for inducing distinct immune responses mediated by bacterial-fungal interactions. Further studies of the mechanisms contributing to these interactions, such as quorum sensing or dectin-1 mediated activation of CD4+ Th2 cells, is necessary for understanding the pathophysiology of CRS and developing microbiome-mediated therapeutics.

## Data Availability Statement

Sequence data for this study have been deposited in the NCBI. Short Read Archive (SRA) under BioProject ID PRJNA610287.

## Ethics Statement

The animal study was reviewed and approved by Northern Arizona University Institutional Animal Care and Use Committee (IACUC).

## Author Contributions

KL was the main investigator of the project and wrote the most part of the manuscript. OK contributed in animal experiments, processing samples, and analyzing the bioinformatics data. IZ and SK contributed in *in-vitro* experiments, *in-vivo* experiments, and processing samples. EC was the corresponding author who contributed in designing the research project, funding, and writing the manuscript. All authors contributed to the article and approved the submitted version.

## Conflict of Interest

The authors declare that the research was conducted in the absence of any commercial or financial relationships that could be construed as a potential conflict of interest.

## Supplementary Material

Figure S1 *In vivo* mouse co-infection experimental scheme. **(A)** acclimatized mice were intranasally infected with designated microbes for each group. Antibiotics were treated with Fluconazole (15 mg/kg) and Augmentin (100 mg/kg) for 5 days for the antibiotic-treated group. All mice were intranasally infected with designated microbes for each group. **(B)** The representation of infected microbes for each group.

Figure S2 Fluorescent *in-situ* hybridization (FISH) observed using confocal laser scanning microscopy (CLSM) confirmed the inhibition of *P. aeruginosa* on *M. sympodialis* and shows the coexistence of *S. aureus* and *M. sympodialis*.

Figure S3 Principal Coordinates Analysis of Unweighted UniFrac distances of the sinonasal microbiota. Two groups of mice, antibiotic-treated or -naive, were intranasally infected with either one species of bacteria, *P. aeruginosa* (light green), *S. aureus* (light red), or fungi, *M. sympodialis* (dark blue), or two species, *M. sympodialis* with *P. aeruginosa* (dark green) or *S. aureus* (dark red). **(A)** represents beta-diversity of antibiotic-treated (solid sphere) and antibiotic-naïve (diamond) mice groups. **(B)** represents beta-diversity of the infection groups in the antibiotic-treated mice, and **(C)** represents phylogenetically weighted beta-diversity of the infection groups in the antibiotic-naïve mice.

Figure S4 Principal Coordinates Analysis of Weighted UniFrac distances of the sinonasal microbiota. Two groups of mice, antibiotic-treated or -naive, were intranasally infected with either one species of bacteria, *P. aeruginosa* (light green), *S. aureus* (light red), or fungi, *M. sympodialis* (dark blue), or two species, *M. sympodialis* with *P. aeruginosa* (dark green) or *S. aureus* (dark red). **(A)** represents beta-diversity of antibiotic-treated (solid sphere) and antibiotic-naïve (diamond) mice groups. **(B)** represents beta-diversity of the infection groups in the antibiotic-treated mice, and **(C)** represents beta-diversity of the infection groups in the antibiotic-naïve mice.

Figure S5 Principal Coordinates Analysis of Jaccard distances of the sinonasal microbiota. Two groups of mice, antibiotic-treated or -naive, were intranasally infected with either one species of bacteria, *P. aeruginosa* (light green), *S. aureus* (light red), or fungi, *M. sympodialis* (dark blue), or two species, *M. sympodialis* with *P. aeruginosa* (dark green) or *S. aureus* (dark red). **(A)** represents beta-diversity of antibiotic-treated (solid sphere) and antibiotic-naïve (diamond) mice groups. **(B)** represents beta-diversity of the infection groups in the antibiotic-treated mice, and **(C)** represents beta-diversity of the infection groups in the antibiotic-naïve mice.

Supplemental Information Relative bacterial abundances in sinonasal microbiota of intranasally infected mice with either bacteria or fungus or both. Available at this link (https://view.qiime2.org/visualization/?type=html&src=https%3A%2F%2Fdl.dropbox.com%2Fs%2F7r7k1c4ridwv8qv%2FDay1-sinus_taxa-barplot.qzv%3Fdl%3D1) or on github at https://github.com/e-cope/malassezia-ms. (Taxonomic level 7, Sort samples by Description and TreatmentGroup). The interactive QIIME 2 analysis file can be viewed at https://view.QIIME2.org/.
