# Supplementary figures and images for: Co-infection of Malassezia sympodialis With Bacterial Pathobionts Pseudomonas aeruginosa or Staphylococcus aureus Leads to Distinct Sinonasal Inflammatory Responses in a Murine Acute Sinusitis Model
Source: Front Cell Infect Microbiol. 2020 Sep 4;10:472. doi: 10.3389/fcimb.2020.00472 (PMC7498577; doi:10.3389/fcimb.2020.00472)

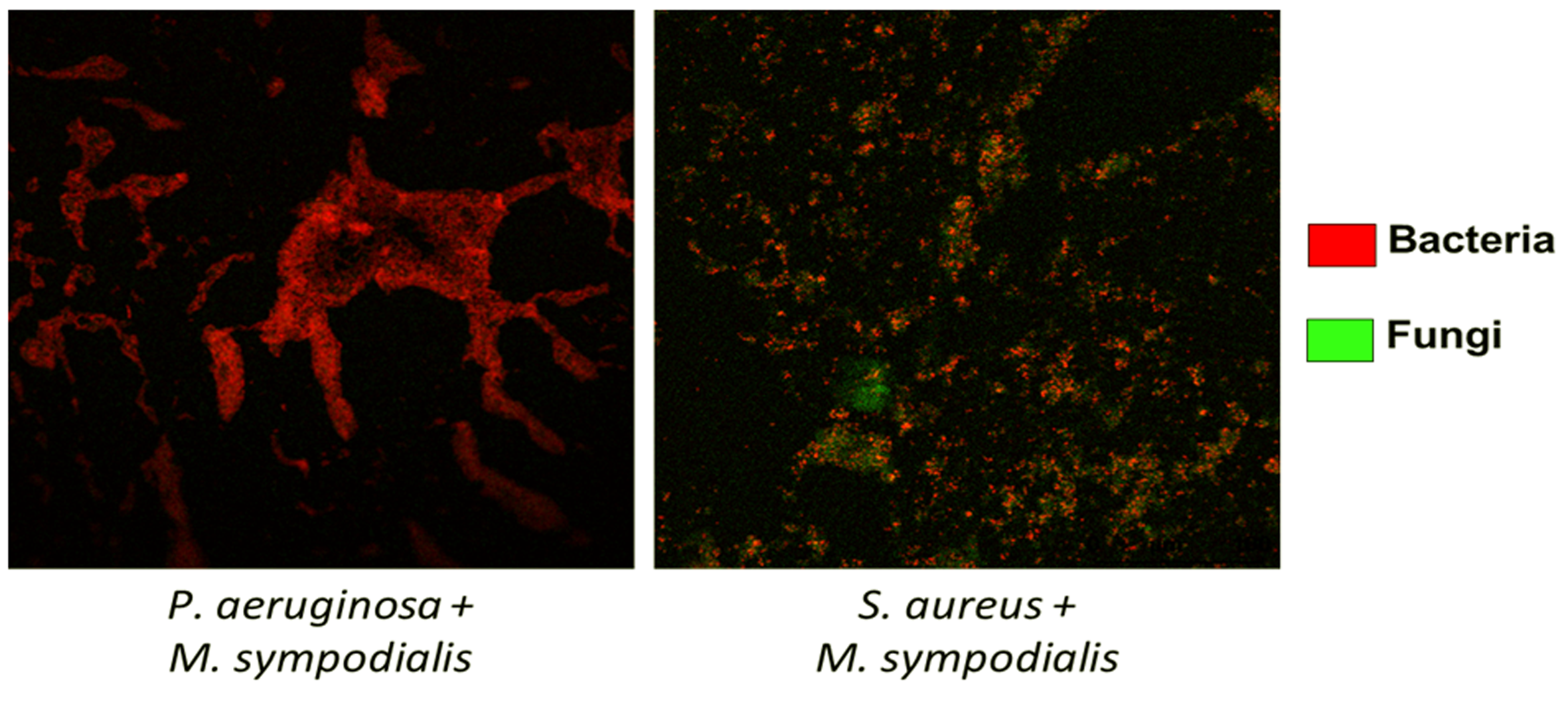

Supplement: Figure S1 — In vivo mouse co-infection experimental scheme. (A) acclimatized mice were intranasally infected with designated microbes for each group. Antibiotics were treated with Fluconazole (15 mg/kg) and Augmentin (100 mg/kg) for 5 days for the antibiotic-treated group. All mice were intranasally infected with designated microbes for each group. (B) The representation of infected microbes for each group. [file Image_1.TIF]

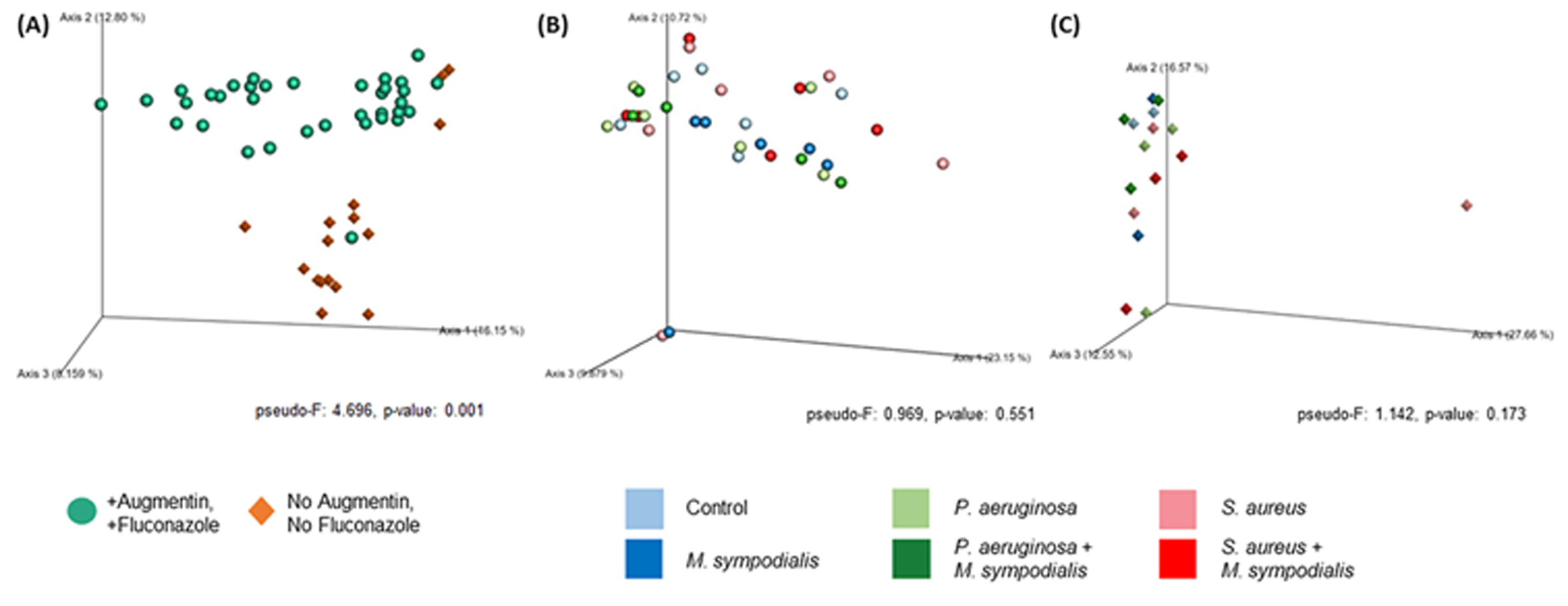

Supplement: Figure S2 — Fluorescent in-situ hybridization (FISH) observed using confocal laser scanning microscopy (CLSM) confirmed the inhibition of P. aeruginosa on M. sympodialis and shows the coexistence of S. aureus and M. sympodialis. [file Image_2.TIF]

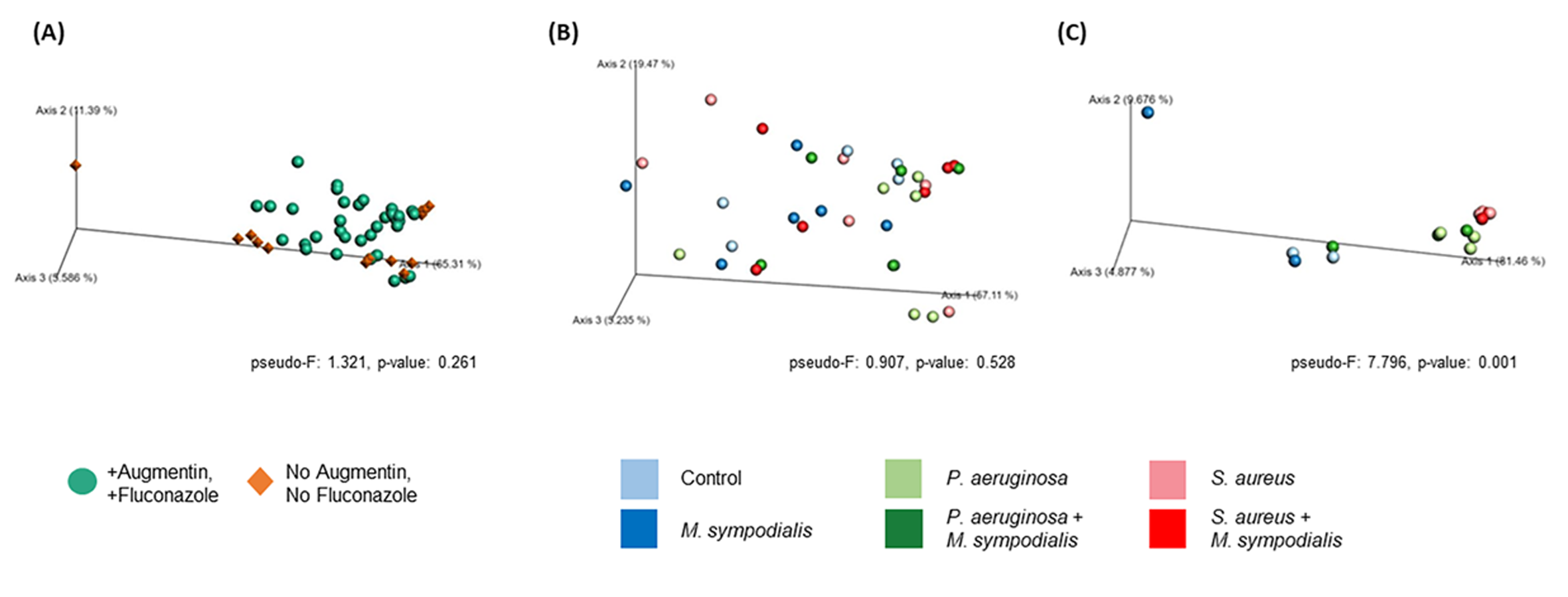

Supplement: Figure S3 — Principal Coordinates Analysis of Unweighted UniFrac distances of the sinonasal microbiota. Two groups of mice, antibiotic-treated or -naive, were intranasally infected with either one species of bacteria, P. aeruginosa (light green), S. aureus (light red), or fungi, M. sympodialis (dark blue), or two species, M. sympodialis with P. aeruginosa (dark green) or S. aureus (dark red). (A) represents beta-diversity of antibiotic-treated (solid sphere) and antibiotic-naïve (diamond) mice groups. (B) represents beta-diversity of the infection groups in the antibiotic-treated mice, and (C) represents phylogenetically weighted beta-diversity of the infection groups in the antibiotic-naïve mice. [file Image_3.TIF]

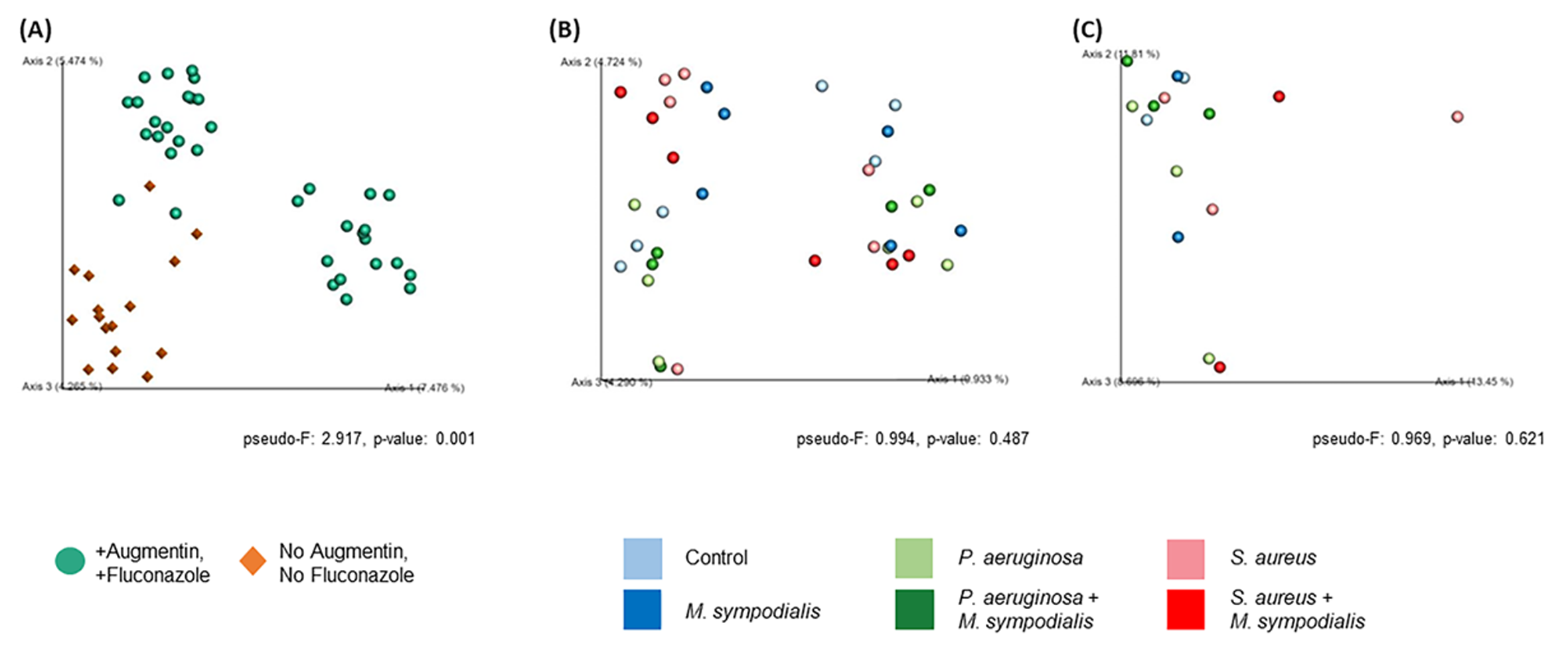

Supplement: Figure S4 — Principal Coordinates Analysis of Weighted UniFrac distances of the sinonasal microbiota. Two groups of mice, antibiotic-treated or -naive, were intranasally infected with either one species of bacteria, P. aeruginosa (light green), S. aureus (light red), or fungi, M. sympodialis (dark blue), or two species, M. sympodialis with P. aeruginosa (dark green) or S. aureus (dark red). (A) represents beta-diversity of antibiotic-treated (solid sphere) and antibiotic-naïve (diamond) mice groups. (B) represents beta-diversity of the infection groups in the antibiotic-treated mice, and (C) represents beta-diversity of the infection groups in the antibiotic-naïve mice. [file Image_4.TIF]

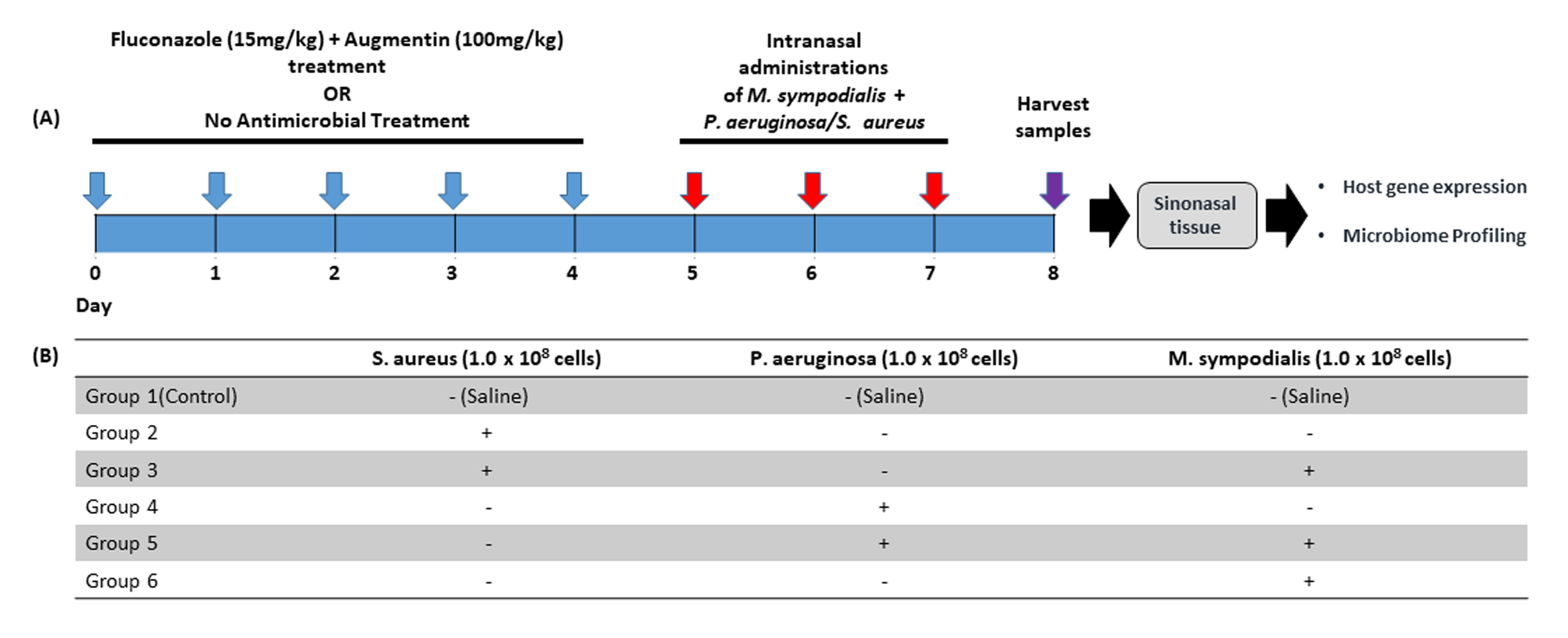

Supplement: Figure S5 — Principal Coordinates Analysis of Jaccard distances of the sinonasal microbiota. Two groups of mice, antibiotic-treated or -naive, were intranasally infected with either one species of bacteria, P. aeruginosa (light green), S. aureus (light red), or fungi, M. sympodialis (dark blue), or two species, M. sympodialis with P. aeruginosa (dark green) or S. aureus (dark red). (A) represents beta-diversity of antibiotic-treated (solid sphere) and antibiotic-naïve (diamond) mice groups. (B) represents beta-diversity of the infection groups in the antibiotic-treated mice, and (C) represents beta-diversity of the infection groups in the antibiotic-naïve mice. [file Image_5.TIF]
